# Plant roots and spectroscopic methods – analyzing species, biomass and vitality

**DOI:** 10.3389/fpls.2013.00393

**Published:** 2013-10-09

**Authors:** Boris Rewald, Catharina Meinen

**Affiliations:** ^1^Institute of Forest Ecology, Department of Forest and Soil Sciences, University of Natural Resources and Life SciencesVienna, Austria; ^2^Division of Agronomy, Department of Crop Sciences, Georg-August-Universität GöttingenGöttingen, Germany

**Keywords:** electrochemical impedance spectroscopy, fine root, IR spectrometry, root biomass, root taxa, root vitality

## Abstract

In order to understand plant functioning, plant community composition, and terrestrial biogeochemistry, it is decisive to study standing root biomass, (fine) root dynamics, and interactions belowground. While most plant taxa can be identified by visual criteria aboveground, roots show less distinctive features. Furthermore, root systems of neighboring plants are rarely spatially segregated; thus, most soil horizons and samples hold roots of more than one species necessitating root sorting according to taxa. In the last decades, various approaches, ranging from anatomical and morphological analyses to differences in chemical composition and DNA sequencing were applied to discern species’ identity and biomass belowground. Among those methods, a variety of spectroscopic methods was used to detect differences in the chemical composition of roots. In this review, spectroscopic methods used to study root systems of herbaceous and woody species in excised samples or *in situ* will be discussed. In detail, techniques will be reviewed according to their usability to discern root taxa, to determine root vitality, and to quantify root biomass non-destructively or in soil cores holding mixtures of plant roots. In addition, spectroscopic methods which may be able to play an increasing role in future studies on root biomass and related traits are highlighted.

## INTRODUCTION

Studying standing root biomass, root dynamics, and interactions belowground is essential for understanding plant functioning, plant community composition, and terrestrial biogeochemistry (de Kroon and Visser, 2003). Root systems of neighboring plants are rarely spatially segregated, thus, most soil sectors of pristine, agricultural, or silvicultural ecosystems hold roots of more than one species (Hutchings and John, 2003). While most plant taxa can be identified by aboveground criteria, such as flower and leaf morphology, roots show less distinctive features. Because root biomass is the major plant parameter governing water and nutrient uptake (Lambers et al., 2008) and a major sink for plants’ carbohydrates, belowground proportions of species have to be quantified, distinguishing living and dead roots. In the last decades, various approaches, ranging from anatomical and morphological analyses to differences in chemical composition and DNA sequencing were applied to discern species identity and biomass belowground (Rewald et al., 2012). Among those methods, spectroscopic methods were used to detect differences in the chemical composition of roots. Furthermore, root biomass is commonly determined by destructive sampling; to allow for continuous measurements and to reduce the costs of labor intensive root washing procedures, fast and non-destructive methods to determine standing (fine) root biomass and biomass increment are needed.

In this review, essential aspects of spectroscopic methods used to study root systems of herbaceous and woody species in excised samples or *in situ* will be discussed. In detail, spectroscopic techniques will be reviewed according to their usability to discern root taxa, to determine root vitality, and to quantify root biomass non-destructively or in soil cores holding mixtures of plant roots. Techniques suitable for root analyses on an ultra-structural scale, such as electron energy loss (EEL) spectroscopy (Watteau et al., 2002), are not addressed by this review while spectroscopic methods which are suggested playing an increasing role in future studies on root biomass and related traits are outlined.

## SPECIES TAXA DETERMINATION

**FIGURE 1:**
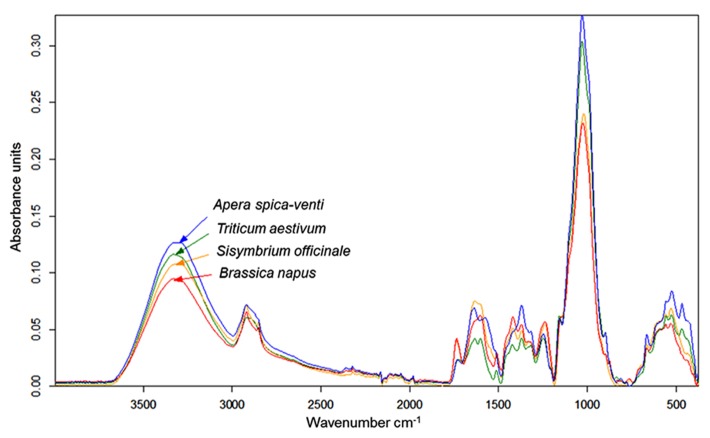
**FT-MIR–ATR spectra recorded from dried, ground roots of *Triticum aestivum* (TA), *Apera spica-venti* (AS), *Brassica napus* (BN), and *Sisymbrium officinale *(SO) grown in a greenhouse experiment.** Spectra are means of four replications, vector-normalized and offset-corrected (C. Meinen, unpublished data).

**FIGURE 2:**
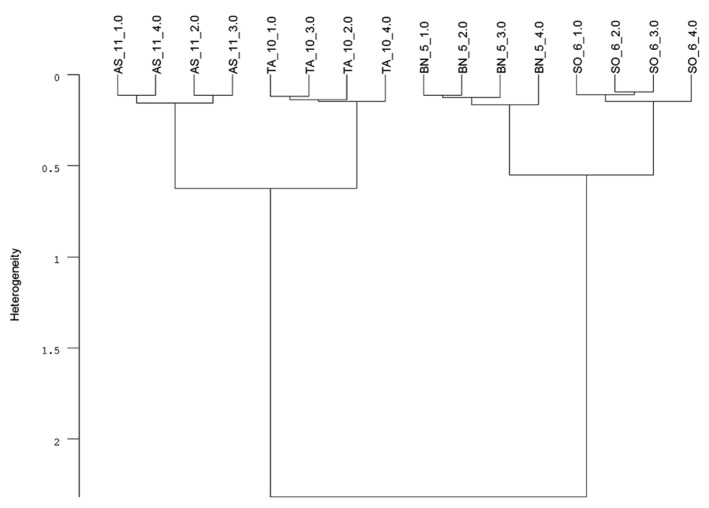
**Cluster analysis of FT-MIR–ATR spectra (**Figure 1**) recorded from dried, ground roots of *Triticum aestivum* (TA), *Apera spica-venti* (AS), *Brassica napus* (BN), and *Sisymbrium officinale *(SO) grown in a greenhouse experiment.** Data were pre-processed by first derivative and vector normalization. Spectral distances were calculated by Euclidean distance and Ward’s algorithm in the frequency range of 374–3999 cm^-^^1^ (mean, *n* = 4; C. Meinen, unpublished data).

Infrared (IR) spectroscopy, especially near-infrared (NIR) and mid-infrared (MIR) spectroscopy, is a standard method to identify and quantify substances. The principle of IR spectroscopy is irradiating a sample and recording the spectral pattern. The chemical composition of a sample determines the spectral print as a function of wavenumber (Chalmers and Griffiths, 2002; Günzler and Gremlich, 2002) and can be utilized for taxa identification. For example, MIR spectroscopy combined with Fourier transformation is able to detect differences in cell-wall composition of leaves which reflect the phylogenetic relationship of plant species (Kim et al., 2004) and was used for species discrimination of fungi and bacteria (Naumann et al., 2005). Today, Fourier transform-infrared (FT-IR) spectroscopy is most commonly used and offers advantages such as short measuring times and high signal-to-noise ratios. While early studies on roots applied IR spectroscopy to determine chemical changes in root tissues of one species (Garrigues et al., 2006; Zhang et al., 2008; Durand et al., 2009). Naumann et al. (2010) achieved a 100% correct discrimination of excised *Pisum sativum* and *Avena sativa* roots by FT-MIR spectroscopy with an attenuated total reflectance (ATR) device. One advantage of MIR, compared with NIR, is the more structural spectra and display of the “fingerprint region” (1500-600 cm^-^^1^) which is highly characteristic for specific substances and, consequently, beneficial for taxa identification (Skrabal, 2009). FT-MIR–ATR spectra of ground roots of *Brassica napus*, *Triticium aestivum*, *Apera spica-venti*, and *Sisymbrium officinale* differ in peak location and peak height (**Figure 1**). Species-specific peaks were especially distinct in the wavenumber region of 1800–400 cm^-^^1^. Cluster analysis of the FT-MIR–ATR root spectra allowed for a complete separation according to species (**Figure 2**). Thus, even the discrimination of closely related crop and weed species within one plant family like *T. aestivum* and *Apera spica-venti* as well as *Brassica napus* and *Sisymbrium officinale* was possible. The spectral differences between monocotyledons and dicotyledons were more pronounced within one taxonomic group and inter-specific differences of these species’ chemical composition were found higher than the intra-specific heterogeneity. The same distinct differences were also found between the closely related species *Zea mays* and *Echinochloa crus-galli* (Poaceae) as well as *Beta vulgaris* and *Chenopodium album* (Amaranthaceae; Meinen and Rauber, 2012). Pronounced differences in spectra are the main requirements to distinguish plants on the level of taxonomic groups or species. However, while Naumann et al. (2010) did not find differences in species’ spectral patterns between different environments (i.e., substrate, competitive neighborhood) and root segment positions, Zuo et al. (2007) divided *Polygonum cuspidatum* root samples from seven geographical origins in China into six classes with principal component analysis (PCA) based on IR fingerprint spectra (Xu et al., 2003). Furthermore, different spectral pattern were detected if roots were inoculated with either rhizobacteria (El Zemrany et al., 2007) or mycorrhizal fungi (Calderón et al., 2009), or treated with environmental pollutants such as benzotriazole (Dokken and Davis, 2011). Thus, because changes in chemical root composition can be caused by changes in abiotic and biotic environments and by secondary growth of roots (White et al., 2011), pure reference samples reflecting the environmental variability and the analyzed “type” of root system are needed to establish more reliable calibration spectra for each species. Beside the need for calibration to local growth conditions, current results demonstrate the ability of IR spectroscopy for distinguishing plant root taxa (**Figure 2**).

**FIGURE 3:**
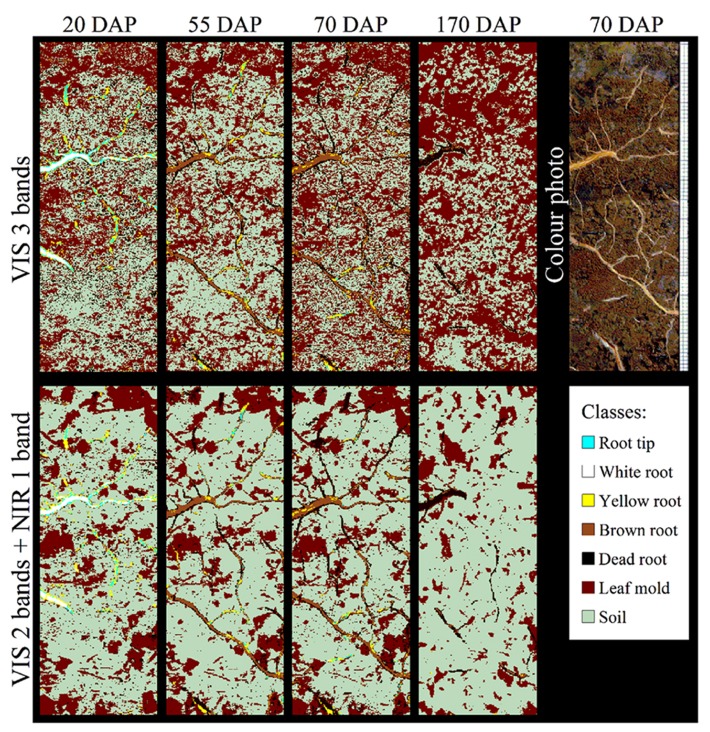
**Classification of *Populus* spp. rhizosphere components using different combinations of visible (VIS) and near infrared (NIR) wave bands to produce spectral reflectance images at days 20, 55, 70, and 170 after planting (DAP).** Wavelengths of the three VIS bands (upper row) and the VIS–NIR bands (lower row) are 650, 550, and 480 nm, and 886, 679, and 522 nm, respectively. A color photo at DAP 70 and the false color classification scheme are given. Source images were taken under wet soil conditions (see Nakaji et al., 2008 for details). Images courtesy of T. Nakaji, K. Noguchi and H. Oguma, Japan.

However, spectroscopic techniques to distinguish taxa have only been applied on excised samples. A non-invasive approach using root windows in combination with visible (VIS) and NIR reflectance spectra to distinguish rhizosphere components and root of varying viability (see below) was successfully tested by Nakaji et al. (2008; **Figure 3**) but did not report species discrimination. Thus, non-destructive approaches for root taxa determination *in situ* are lacking to date although non-invasive, “remote” IR spectroscopy is common in applied fields such as plastic waste identification (Wienke et al., 1995) and clinical tissue oxygen analyses (Crespi, 2007). While non-invasive IR techniques have the potential to dramatically enhance the application range of (mini-)rhizotrons in mixtures, allowing for species-specific root growth analyses (Pierret, 2008; Rewald and Ephrath, 2013), the different water contents of fresh roots are currently restricting its application. Due to the strong dipole moment of water, which results in a strong signal, applications of IR spectroscopy were for long focused on dry material. Thus an advantage of the ATR techniques is the usability of fresh root material without sample preparation like drying, grinding, or potassium bromide (KBr) pellets. In addition, ATR techniques require only small amounts of sample material which is in many cases limited in root studies. Recently, Meinen and Rauber (2012) noted that fresh rootlets spectra of closely related species (i.e., maize, barnyard grass) showed similar peak distribution when analyzed by FT-MIR–ATR and could not be discriminated by cluster analysis whereas dry rootlets differed in peak location and height. However, distantly related species such as pea and oat could be discriminated by utilizing fresh samples (C. Meinen, unpublished results). While the susceptibility of IR spectroscopy to water content is an intrinsic phenomenon of this technique, a probable solution for closely related species could be the future use of Raman spectroscopy (see below).

## TOTAL AND TAXA-SPECIFIC ROOT BIOMASS QUANTIFICATION

**FIGURE 4:**
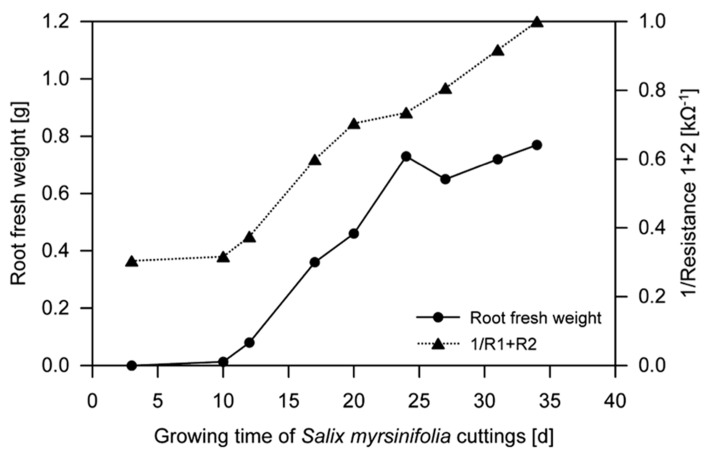
**Fresh weight of hydroponically grown *Salix* spp. roots and the reciprocal sum of resistances R_**1**_ and R_**2**_ during growth (mean, *n* = 3; Repo et al., 2005, modified).** Electrochemical impedance spectroscopy (EIS) was used to determine R_1_ and R_2_ (see Repo et al., 2005 for details).

Dielectric spectroscopy/electrochemical impedance spectroscopy (EIS) methods are widely used to investigate the properties of soils as well as plant and animal tissues (Repo et al., 2012). EIS studies the response to the application of a periodic small amplitude alternating current (AC) signal; measurements are carried out at different AC frequencies and an impedance spectrum is recorded (Lasia, 1999; Cao et al., 2011). A general principle of EIS is that the (root) system is exposed to an alternating electric field that would cause polarization and relaxation phenomena. EIS methods are regarded as an enhancement of earlier electrical measurements at a single frequency (e.g., root capacitance, impedance, and potential; Urban et al., 2011). They have the potential to study (fine) root biomass by the proxy of “active,” i.e., conductive, root surface area (see Repo et al., 2012 for review). Ozier-Lafontaine and Bajazet (2005) used 51 frequencies between 10 Hz and 1 MHz to measure the impedance spectra of soil-grown *Solanum lycopersicum*. A coefficient of determination of 0.98 was found between the models, based on complex non-linear least squares (CNLS) curve-fitting and including the model variables capacitance C_3_ and C_4_, and the increment in root dry weight (see Ozier-Lafontaine and Bajazet, 2005 for details). The high correlation was suggested to be likely based on the proportionality between root capacitance and the quantity of root cells/membranes. In contrast, resistance was found to be the main relevant variable for root length determination, permitting the possibility to distinguish between root biomass and length using EIS. In the same year, Repo et al. (2005) used EIS in a frequency range from 40 Hz to 340 kHz to determine the root biomass of hydroponically grown *Salix* clones. The most important finding was that the sum of the resistances R_1_ and R_2_ (i.e., the resistor of the distributed ZARC-Cole element (Barsoukov and Macdonald, 2005)) in the developed electric model decreased with an increase in root mass of each individual plant (**Figure 4**). These studies encourage further research on the applicability of EIS methods as a fast way to determine root growth and root biomass of single plant individuals with rather minor destructions (i.e., electrode installation in the shoot). However, future studies are required since environmental parameters such as soil type, and soil and root moisture contents, known to have strong effects on capacitance (Dalton, 1995; Repo et al., 2000), and the influence of the experimental set-up, especially electrode position (Repo et al., 2012), have not been fully understood yet.

While useful for non-destructive root biomass estimation of single plant individuals with sufficiently sized stems, EIS cannot be directly used to determine the proportion of species’ roots in plant mixtures. However, understanding plant community functioning and geochemical consequences (e.g., in terms of C sequestration) requires an accurate assessment of the belowground biomass and the distribution of each species in the community. Similar to the procedure of taxa determination by IR spectroscopy (see above), differences in the chemical composition of mixed root samples can be utilized to determine biomass proportions in excised soil samples. Aboveground, IR spectroscopy has been frequently used to determine the botanical composition of forage mixtures (Coleman et al., 1985, 1990). On roots, NIR spectroscopy was applied first by Rumbaugh et al. (1988) to predict the root biomass proportion of four grass species in binary mixtures with *Medicago sativa*. Prepared *Medicago*-grass root mixtures with grass ratios from 0 to 1 were recorded by NIRS, and root spectra were correlated to corresponding root proportion. Coefficients of determination ranged from 0.92 to 0.99 between the actual and the predicted root mass ratio. Similarly, Roumet et al. (2006) tested artificial root mixtures with two and three species (five Poaceae, one Asteraceae), and estimated the proportion of the species in the mixtures using NIR spectra (25000–4000 cm^-^^1^) with high accuracy (*r* = 0.99). Finally, the prediction of species ratios in woody fine-root mixtures (*Fagus sylvatica*, *Quercus petraea*, *Picea abies*, *Pseudotsuga menziesii* – with two, or up to four tree species plus herbal roots) was successfully demonstrated by Bauhus and colleagues (Bauhus et al., 2007; Lei and Bauhus, 2010). Even in tree root samples with low abundance of specific species, NIR models presented reasonable approximations of the biomass abundance.

To determine the species composition in mixed samples with IR spectroscopy, reference samples of pure material are imperative to create calibration series. Calibration samples are created by mixing pure (ground) material of the species for which the calibration is developed in known proportions and continuously. Roumet et al. (2006) suggested that each calibration required the preparation of >40 artificial mixtures. On fodder mixtures, two different methods have been applied to provide the pure material for calibration series, so called “artificial” and “real” samples (Cougnon et al., 2013). It has been claimed that calibrations based on “artificial” samples, which are attained from pure stands (Coleman et al., 1985; Petersen et al., 1987), have good calibration statistics but fail to predict real validation samples while “real” samples, obtained by hand sorting of species mixtures (Shaffer et al., 1990; Wachendorf et al., 1999), reflect the environmental and thus spectral variation to a greater extent (Pitman et al., 1991; Cougnon et al., 2013). Studying roots, Roumet et al. (2006) used both “artificial” and “real” samples, originating from both natural and environmentally controlled conditions, and found that the predictive equations for calibration were robust. However, the impact of the calibration material origin has not been addressed in greater depth and additional research is necessary. However, regarding the difficult determination of root taxa by visual criteria, which makes hand sorting of grass and herbaceous roots impossible (Rewald et al., 2012), and the high amount of pure sample material needed (~15 g dry weight per species; Roumet et al., 2006), the use of pure “artificial” samples grown under similar environmental conditions as the analyzed sampled might be the only practical and economically feasible option.

In contrast to other studies, which utilized only one bulk sample for each species to generate artificial mixtures (e.g., Roumet et al., 2006), the mixed samples created by Lei and Bauhus (2010) also captured the variation in spectral properties that occur within each species. To capture this variation is very important if species proportions should be predicted in field samples. The quality of prediction of NIR models was assumed to decline with increasing species numbers within samples (Coleman et al., 1990). However, Lei and Bauhus (2010) could show that models based on three components are not more robust than models based on five species. Furthermore, even in highly species diverse forest ecosystems, a single tree has a limited number of inter-specific neighbors. Thus at any given point in space, the actual number of species contained in fine-roots samples from forests will be limited. The same holds true for agricultural mixtures while further research is needed for ecosystems with a high diversity at a small scale such as grasslands. Similar to NIR models, MIR models can predict species proportions in mixtures of herbaceous species. To study the impact of weed roots, a Fourier transform MIR–ATR model for *Vicia faba* and *Matricaria chamomilla* was developed (**Table 1**). Model development and data analysis were carried out with the software OPUS QUANT 2 (Version 7.0; Bruker, 2011). The cross-validation option was used to create the model. The best model with highest coefficient of correlation (*R*^2^) and lowest root mean square error of cross validation (RMSECV) was selected by running a procedure for model optimization provided by the software. An independent sample set (*n* = 16) with known species proportion was used for external validation. This validation revealed a high correlation coefficient (0.99) and a low RMSE of prediction (4.43). Even with a relatively small number of 21 calibration samples the quality of the model was very good. These results indicate that MIR models are a promising tool to quantify species proportions in root mixtures of herbaceous plants.

**Table 1:** Statistical parameters of the two-component FT-IR–ATR model for *Vicia faba* and *Matricaria chamomilla* in terms of calibration, validation and external test set validation.

Model	Calibration (*n* = 21)		validation				External validation			
	*R*^2^	RMSEE	*R*^2^	RMSECV	Bias	RPD	*r*	RMSEP	RPD	Outlier
*Vicia–Matricaria*	98.46	3.91	98.04	4.19	±0.50	7.15	0.99	4.43	6.19	0

*Model quality is described by coefficient of determination (*R*^2^), root mean square error of calibration (RMSEE), root mean square error of cross validation (RMSECV), residual predictive deviation (RPD), correlation coefficient (r), and root mean square error of prediction (RMSEP). Spectra were min–max normalized and the wavenumber region of 3997.3–2185 cm ^-^^1^ and 1823.6–735.4 cm ^-^^1^ were considered. An optimization procedure was used to select the best model (C. Meinen, unpublished data).*

Thus, IR spectroscopy can accurately estimate the botanical composition of moderately diverse root mixtures if sufficient amounts of pure root material are available for calibration. The roots for calibration should be grown and harvested under the same conditions as those in mixed samples.

## ROOT VITALITY

The ability to determine the vitality of roots, with its extremes “life” and “dead,” is of utmost importance to identify the active (fine) root biomass available for water and nutrient uptake and to determine root longevity. IR spectroscopy has been used to distinguish alive, injured and dead bacteria (Davis and Mauer, 2010) and to discriminate damaged seeds (Agelet et al., 2012) with varying success (see below).

Picon-Cochard et al. (2009) used NIR spectroscopy to predict the percentage of dead versus living roots of five grass species grown in monocultures under field conditions. Root death was induced after total severance of aboveground vegetation. Root samples were collected immediately after this treatment to obtain predominantly live roots, and then 1 and 2 months later to obtain dead roots. NIR spectra of Control samples were different from later samples for four of the five species. The percentage of live and dead roots was significantly predicted by NIR with an error of prediction of 15%. Working in forests, Bauhus et al. (2007) stated that the proportion of live and dead *F. sylvatica* and *Picea abies* fine roots can be determined with high accuracy using NIR analysis. The two studies show the potential of NIR to predict the percentage of dead and live roots in excised samples. In the field, Nakaji et al. (2008) used VIS and NIR reflectance spectra of glass-faced rhizoboxes to automatically distinguish *Populus* spp. roots from soil and leaf mold and to classify roots into four age classes and dead roots (**Figure 3**) as reported earlier for crop residue (Daughtry et al., 1995). An ethylene vinyl acetate card was used by Nakaji et al. (2008) to standardize their reflectance measurements, an approach likely similar to the calibration model developed by Watari and Ozaki (2005). The reflectance of dead roots was lower than that of mature roots in both VIS and NIR spectral regions. Although the most suitable spectral bands differed between moist and dry soil conditions, and fewer pixels were classified correctly in dryer soil, the spectral bands 17200-14700 and 11800-11200 cm^-^^1^ provided reasonable reliability under both conditions. Classification accuracy was higher when using two to five VIS–NIR images (overall accuracy ≤87.8%) than three VIS images (red, green, and blue; accuracy <67.1%). Irrespective of soil moisture condition, the overall accuracy tended to be stable at 92–94% with use of four to five VIS–NIR wavebands; however, the same accuracy could not be obtained for all age classes. The spectral bands effective under wet soil conditions could also be used for classification in dry conditions, with overall accuracies >86.9%. These results suggest that automatic image analysis using combined VIS–NIR images of multiple spectral bands (**Figure 3**) allows for accurate classification of *Populus* spp. roots’ live/dead status and relative accurate classification of the age class. While further studies on roots are absent, it is known for bacteria and seeds that spectral differences between live and injured bacteria or heat and freezing stress can be hardly discernible due to the minor compositional differences (Davis and Mauer, 2010; Agelet et al., 2012). Thus, Davis and Mauer (2010), for example, used second derivative pre-processing to increase the number of discriminative features and PCA and soft independent modeling by class analogy (SIMCA) models to correctly classify live and injured bacteria. Similar procedures might be applicable for root vitality analyses with spectroscopic techniques. Further studies on additional species, growing in different soil types and under a larger range of soil moisture conditions are needed to determine the full capacity of VIS–NIR spectroscopy for root viability determination.

## SUITABILITY OF OTHER SPECTROSCOPIC TECHNIQUES FOR ROOT ANALYSES

### FLUORESCENCE SPECTROSCOPY

Fluorescence is the emission of light subsequent to absorption of ultraviolet (UV) or VIS light by a fluorescent molecule or substructures called fluorophore. Thus, the fluorophore absorbs energy in the form of light at a specific wavelength and emits energy in the form of light emitted at a lower energy level. Fluorescence spectroscopy (FS) is widely used for chemical analyses of auto-fluorescing molecules (Sharma and Schulman, 1999); in plants, chlorophyll fluorescence has been widely used to determine the physiological status of leaves (Zinnert et al., 2013). While *in situ* root observations on *Glycine max* showed that nutrient absorption and root elongation rates positively correlate to fluorescence intensity (Dyer and Brown, 1983) and that fluorescence can be influenced by microbial colonization (Gamalero et al., 2004) non-species-specific influences are questioning the applicability of simple fluorescence measurements for root classification. However, more advanced FS approaches obtaining information about the entire fluorescence landscape (or at least >2 excitation wavelength and spectra of emission) in order to find excitation and emission maxima as well as the structure of the curve are likely to allow the application of FS on roots. The utilization of FS is of particular interest because a combined fluorescence and reflectance spectroscopy approach on animal tissues was found to significantly improve signal/noise ratios, disentangling absorption, scattering, and intrinsic fluorescence parameters (Müller et al., 2002).

### ENERGY DISPERSIVE X-RAY SPECTROSCOPY

A variety of X-ray spectroscopic methods has been used to characterize plant tissues in the past as well as plant–environmental interactions such as the biochemistry of the rhizosphere. For example, Agrahari et al. (2010) successfully determined the chemical properties of dried, powdered root tubers with energy dispersive X-ray (EDX) spectroscopy. While EDX has not been used to discriminate among root taxa yet, EDX spectra reflect the composition of the tissue and might thus be used for such purposes similar to IR spectrometry (see above). Frommer et al. (2011) and others used (micro-focused) energy-dispersive X-ray fluorescence (XRF) spectrometry to determine the spatial distribution of certain metal elements. It seem feasible to use this technique to determine plant root taxa in soil thin sections prepared from undisturbed soil samples, although the work load for sample preparation is high and the method is not amendable to study many samples.

### RAMAN SPECTROSCOPY

Raman spectroscopy (RS) is in general less widely used than IR spectroscopy, mainly due to early difficulties with sample degradation and fluorescence (Allison, 2011). However, a renaissance of Raman spectroscopy is triggered by advanced laser technology, by efficient interference filters to suppress elastically scattered Rayleigh light, by the development of more sensitive detectors and new methodological approaches (e.g., FT-RS; Schmitt and Popp, 2006; Schulz, 2008). These improvements, together with the ability to examine raw samples without any preparation by FT-RS have led to a rapid growth in application (Kizil and Irudayaraj, 2008). While FT-RS has been successfully used as a non-destructive technique for chemotaxonomy on aboveground tissues (Schrader et al., 1991, 1999) and to determine concentrations of specific chemicals within roots (Andreev et al., 2001; Frosch et al., 2007; de Oliveira et al., 2009) it has, to the best of our knowledge, not been used to discriminate among root taxa. Compared to IR spectrometry, RS has two major advantages which make this technique most suitable to perform *in situ* studies on roots, (i) water content has only weak Raman scattering properties (Gierlinger and Schwanninger, 2007), and (ii) RS does not require optically clear samples making measurements through colored glass and plastic feasible – which are used at the soil interface in (mini-)rhizotron studies (Rewald and Ephrath, 2013).

### NUCLEAR MAGNETIC RESONANCE SPECTROSCOPY

Nuclear magnetic resonance (NMR) spectroscopy is a non-destructive analytical method that generates data on the presence of a wide range of low molecular weight metabolites in aqueous extracts. Several studies have used NMR-based methods on plant material, demonstrating biochemical differences according to tissue age, geographical location, genetic modification, and response to stress. For example, Shin et al. (2007) performed fingerprinting analyses of fresh, differently old ginseng roots using ^1^H-NMR spectroscopy and multivariate analysis techniques. The authors detected various distinct peaks in the ^1^H-NMR spectra of the liquid-state ginseng roots. Two dimensional score plots of a PCA showed clear separations of components at different roots ages, and explained 89.6% of the total variance. These results and others indicate that the combination of ^1^H-NMR and PCA provides a powerful tool for authenticating root taxa, differentiating between root age classes and to investigate the effects of several environmental stressors (Gavaghan et al., 2011). Other NMR spectroscopic approaches such as ^14^N-NMR or ^13^C-NMR, and especially those allowing for solid state analyses, could be similarly suitable to distinguish plant individual or taxa and to determine root viability by metabolic profiling (Farag et al., 2012; Incerti et al., 2013). While solid state (^13^C)-NMR spectroscopy should be especially useful, its availability is limited to, so far, only few laboratories. Similar, the laborious preparation of liquid extracts and the high costs of NMR in general will limit the broad use of these techniques for non-molecular analysis and prevents high throughput analysis of root samples.

## CONCLUSION AND OUTLOOK

Internal and diffuse external reflectance techniques based on IR are relatively fast and affordable and proved to be able for distinguishing even closely related species, quantifying the biomass proportion of species in mixtures and/or determining the root viability status. However, a plethora of other spectroscopic techniques is available but yet rarely used for these purposes. Thus even though most of the techniques mentioned above provide a powerful tool for the investigation of roots, a most accurate determination can likely be obtained through the integration of complementary approaches. Especially the usability of FT-RS and fluorescence spectrometry should be evaluated further because of the ability of these techniques to analyze roots through glass/plastic windows – as commonly used for (mini-)rhizotron studies – possibly overcoming uncertainties of earlier UV–VIS-based methods to determine root vitality (Wang et al., 1995). Beside the actual techniques, the broad identification of root taxa of single roots and in root mixtures and the determination of viability parameters is hampered by the availability of databases of species- and environmental-specific spectral profiles of roots. This would help researchers to identify even unknown roots in their sample, similar to gene sequence databases for root research (Rewald et al., 2012).

## Conflict of Interest Statement

The authors declare that the research was conducted in the absence of any commercial or financial relationships that could be construed as a potential conflict of interest.
